# How will southern hemisphere subtropical anticyclones respond to global warming? Mechanisms and seasonality in CMIP5 and CMIP6 model projections

**DOI:** 10.1007/s00382-020-05290-7

**Published:** 2020-05-10

**Authors:** Abdullah al Fahad, Natalie J. Burls, Zachary Strasberg

**Affiliations:** 1 Department of Atmospheric, Oceanic, and Earth Sciences, George Mason University, Fairfax, VA 22030 USA; 2 James Madison University, Harrisonburg, VA USA

**Keywords:** Subtropical anticyclone, Atmospheric circulation, High pressure system, Climate change, CMIP5, CMIP6

## Abstract

The anticyclonic high-pressure systems over the southern-hemisphere, subtropical oceans have a significant influence on regional climate. Previous studies of how these subtropical anticyclones will change under global warming have focused on austral summer while the winter season has remained largely uninvestigated, together with the extent to which the dominant mechanisms proposed to explain the multi-model-mean changes similarly explain the inter-model spread in projections. This study addresses these gaps by focusing on the mechanisms that drive the spread in projected future changes across the Coupled Model Intercomparison Project Phase 5 and 6 archives during both the summer and winter seasons. The southern hemisphere anticyclones intensify in strength at their center and poleward flank during both seasons in the future projections analyzed. The inter-model spread in projected local diabatic heating changes accounts for a considerable amount of the inter-model spread in the response of the South Pacific anticyclone during both seasons. However, model differences in projected zonal-mean tropospheric static stability changes, which in turn influence baroclinic eddy growth, are most influential in determining the often-strong increases in sea level pressure seen along the poleward flank of all the anticyclones during both seasons. Increased zonal-mean tropospheric static stability over the subtropics is consistent with the poleward shift in Hadley cell edge and zonal-mean sea level pressure increases. The results suggest that differences in the extent of tropical-upper-tropospheric and subtropical-lower-tropospheric warming in the southern hemisphere, via their influence on tropospheric static stability, will largely determine the fate of the anticyclones over the coming century.

**Electronic supplementary material:**

The online version of this article (10.1007/s00382-020-05290-7) contains supplementary material, which is available to authorized users.

## Introduction

**Fig. 1:**
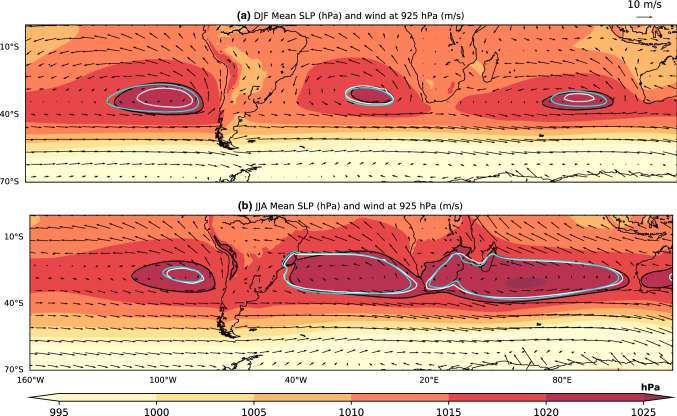
Era-Interim (1979–2016) climatology for **a** DJF and **b** JJA seasonal mean SLP (shaded) (unit: hPa), and 925 hPa winds (vector) (unit: m/s). The 1020 hPa SLP isobar of the Era-Interim is contoured in black, the CMIP5 Historical MMM contoured in white, and the CMIP6 Historical MMM contoured in blue to highlight the climatological position of each SH SA

Subtropical Anticyclones (SAs), also known as Subtropical Highs, are semi-permanent high-pressure systems that cover 40% of the Earth and are centered around 30° latitude in both the Northern Hemisphere (NH) and Southern Hemisphere (SH). While the NH supports only two permanent SAs over its subtropical ocean basins, the North Pacific SA and North Atlantic SA, the SH supports three SAs, the South Pacific SA (SPSA), South Atlantic SA (SASA), and South Indian SA (SISA) (Fig. 1, Fig. S1). SAs are an essential part of the large-scale atmospheric circulation that connects the midlatitude westerly and tropical easterly circulation regimes. Subtropical midlatitude weather and climate in both hemispheres is tightly linked to the nature of the SAs through their influence on local Sea Surface Temperature (SST), precipitation, and the subtropical ocean gyres. SAs play a significant role in regional precipitation by affecting moisture transport between the midlatitude and tropical regions and hence regional precipitation variability over East Asia and Southern USA (e.g. Gamble et al. 2008; Zhou et al. 2009), South America (Brazil, Peru) (e.g. Doyle and Barros 2002; Reboita et al. 2010), Southern Africa (e.g. Burls et al. 2019). Understanding how SAs will respond under global warming condition is a crucial element of future climate projections for these regions.

SAs primarily owe their existence to the descending branch of the Hadley Cell in each hemisphere. Hadley circulation is strongest in the winter hemisphere, with enhanced poleward energy transport from the warm tropics to the colder hemisphere. The strength of the winter hemisphere’s Hadley cell also depends on the strength of heating over the summer hemisphere’s continents (Lee et al. 2013). Given that the descending branch of each Hadley cell is at its strongest during local winter, one would expect the SAs to be reach their maximum strength during December-January–February (DJF) in the NH, and June–July–August (JJA) in the SH. This is not however the case for the NH. Generally speaking, the SAs reach their climatological maximum in both area and strength during local summer in the NH and local winter in the SH (Hoskins 1996; Rodwell and Hoskins 2001; Seager et al. 2003; Nigam and Chan 2009).

This distinct behavior of the NH SAs points to the influence of additional processes controlling the strength of the SAs and has provided the motivation for several studies. Hoskins (1996) first highlighted that one of these processes is monsoonal heating over continents to the east of an ocean basin. The associated diabatic heating generates a Rossby wave response that leads to descent north-west of the monsoonal heating and therefore enhances the strength of the SA over the eastern side of the given ocean basin. Later Rodwell and Hoskins (2001) and Chen et al. (2001) showed monsoonal heating on both continents surrounding a subtropical ocean contribute to the strengthening of SAs. Wu and Liu (2003) show that diabatic heating differences over land and ocean play a significant role in the development and intensification of summer SAs by creating subsidence over the eastern flank of the ocean and ascent over the adjacent continents. As a result, even though the zonal mean component is weaker during NH summer due to weak Hadley cell descend, strong Asian and North American monsoonal heating creates a strong zonally asymmetric component in subtropical Sea Level Pressure (SLP) that leads to North Pacific SA and North Atlantic SA summer maxima (Hoskins 1996; Wu and Liu 2003; Nigam and Chan 2009). In contrast, summer monsoonal heating over the continents on the SH is not as strong as NH summer monsoonal heating and therefore maximum SH Hadley cell descent during JJA leads to the seasonal maximum area and intensity of SH SAs. One exception is the SPSA which is in fact stronger in DJF compared to JJA (Fig. 1, Fig. S1). Previous studies have not discussed this feature before, and we are preparing a follow-up article focusing on this unique property of the SPSA.

For winter SAs, Rodwell and Hoskins (2001) argue that local orography helps to shape SA location, especially in the SH. Richter et al. (2008) show that the descending area of the SH SAs (specifically the SASA and SISA) during local winter (JJA) is simulated best by climate models that simulate the major NH local summer monsoons well, given the influence of NH major summer monsoon heating on SA’s in the SH (Lee et al. 2013).

Recent studies, using observed and reanalysis datasets, show that there is a significant SLP trend in recent decades in the SH subtropical regions (Gillett et al. 2003; Gillett and Stott 2009; Grise et al. 2018; Staten et al. 2018; Vizy et al. 2018; Burls et al. 2019). The area and intensity of the SH SAs have increased in strength and extended towards the pole during both summer and winter seasons. This positive SH subtropical SLP trend is largely attributed to the expansion of the tropical circulation belt and poleward shift of the Hadley Cell edge due to tropical upper tropospheric warming and high latitude lower stratospheric cooling (Gillett and Stott 2009; Grise et al. 2018).

Several studies have investigated the impact of global warming on SA properties in both hemispheres during local summer in future climate projections (Li et al. 2012, 2013; He et al. 2017). Li et al. (2013) evaluated subtropical stream functions in ERA-40 reanalysis data compared with Coupled Model Intercomparison Project Phase 3 (CMIP3) and Phase 5 (CMIP5) multi model projections to show that all three summertime SH SAs are likely to move poleward and intensify over the SH subtropical oceans during DJF. They find that the increased area and intensity is due to increased land-sea thermal contrast, characterized by increased longwave cooling over the eastern flank of the subtropical ocean basin and increased sensible and latent heating over the adjacent continents. Using an idealized general circulation model (IGCM), Li et al. (2013) show that prescribing the diabatic heating changes seen in RCP8.5 relative to Historical over the subtropical oceans and surrounding lands produces similar SH SA stream function changes in the IGCM as in the CMIP Multi Model Mean (MMM). Li et al. (2013) also suggest that local feedbacks between the SAs and marine boundary layer clouds can contribute to summer SH SA changes in a manner consistent with the current understanding of the dynamical forcings of summer SAs.

Based on changes in subsidence at 700 hPa, and focusing on the equatorward flank (10°–40° latitude), He et al. (2017) find that the summer SH SAs (SASA and SISA) weaken in the RCP8.5 MMM (2050–2099) compared to Historical (1950–1999). He et al. (2017) diagnose the relative contribution of changes in horizontal temperature advection, adiabatic heating, and diabatic heating to local changes in the steady state temperature equation. Their analysis suggests that zonal mean tropospheric static stability and local diabatic heating changes are the dominant mechanisms leading to the SA changes in a warming climate. He et al. (2017) attribute the weakening seen along the equatorward flanks of the SH SAs to zonal mean static stability increases following the “positive mean advection of stratification change (MASC)” mechanism of Ma et al. (2012).

In addition to studies that have investigated the mechanisms supporting changes in the SH SAs (the zonally asymmetric component), there is a large body of literature on the mechanisms driving changes in the zonal mean component, specifically Hadley Cell expansion (Tandon et al. 2013; Waugh et al. 2015; Staten et al. 2018). Previous studies have shown that an intensification of the equator to pole temperature gradient on the poleward edge can lead to a poleward shift in baroclinic instability and the zonal-mean Hadley Cell edge (Lu et al. 2008). Using the Phillips Criterion metric for baroclinic instability, Lu et al. (2008) show that the SH Hadley Cell edge shifts poleward when there is a decrease in baroclinic eddy growth under global warming conditions. Due to high latitude lower stratospheric cooling and tropical upper tropospheric heating, static stability increases, stabilizing baroclinic eddy growth on the equatorward flank of the storm tracks. As a result, the decreased baroclinic eddy growth can be viewed as leading to the poleward shift in the Hadley cell in SH summer (Lu et al. 2008). Recently, Song et al. (2018a, b) show that simulated future changes in the zonally symmetric component of the NH SAs largely depends on the seasonal delay of the northward shift of tropical rainfall belts from spring to summer in a warmer climate.

While these studies focus on summer, little research has been conducted on the behavior of the winter SH SAs in a warming climate. The focus of this study is to analyze both the summer (DJF) and winter (JJA) SLP and anticyclonic wind changes associated with the SH SAs in future global warming scenarios from both CMIP5 (RCP 8.5) and CIMP6 (SSP585). As reviewed above, previous studies have identified three dominant mechanisms that could drive SH SA changes: local diabatic heating changes (Li et al. 2012; He et al. 2017); zonal-mean static stability changes via the MASC mechanism (He et al. 2017); and changes in the large-scale conditions promoting baroclinic eddy growth (Lu et al. 2008). Here we evaluate the extent to which the variables associated with these mechanisms change within the MMM of the chosen CMIP5 and CMIP6 scenarios, as well as their ability to explain the CMIP inter-model spread of SH SAs strength change in both SH summer (DJF) and winter (JJA). The layout of this study is structured as follows: Sect. 2 provides a description of the methods used; Sect. 3 the results of our analysis broken down into two main subsection, the first broadly describe the simulated changes in both CMIP5 and CMIP6 and the second investigate the relationship with the mechanisms proposed above; Sect. 4 presents the summary and conclusions of this study.

## Methodology

In this study, the CMIP5 and CMIP6 Historical experiments forced by observed historical forcing are taken to represent present-day climate (years 1950–1999) and are compared with the CMIP5 representative concentration pathway 8.5 (RCP8.5) experiment (years 2050–2099) and the CMIP6 ScenarioMIP SSP585 experiment (years 2050–2099). The ensemble-mean (when more than one realization is available) for each of the 21 CMIP5 models (Supplementary Table S1) and 12 CMIP6 models (Supplementary Table S2) is analyzed. Given that 68% inter-model consistency, if the models were independent of each other, is equivalent to 95% statistical significance determined by a student t test (Power et al. 2012; He et al. 2017), the MMM change of a variable is stippled at each grid point where at least 75% (to make the 21 CMIP5 and 12 CMIP6 models sample critical value somewhat strict) of the models agree on the change of sign. The correlation maps are stippled at the 95% significance level. The CMIP6 archive correlation maps are calculated based on available data as some models have missing data points, especially in CMIP6 DJF over the SH high latitudes. The reanalysis dataset Era-Interim (Dee et al. 2011) is used in this study to compare the climatological position of SAs with CMIP5 and CMIP6 historical projections.

**Fig. 2:**
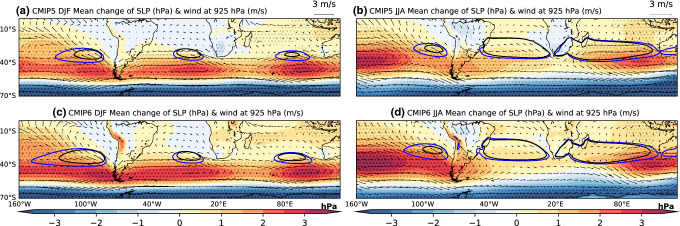
MMM change (Future projection – Historical) of CMIP5 **a** DJF and **b** JJA, and CMIP6 **c** DJF and **d** JJA seasonal mean of SLP (shaded; stippled at 95% significance level) (unit: hPa), and wind at 925 hPa (vector grey) (unit: m/s). The 1020 hPa Historical SLP isobar is contoured in black and the future projection isobar is contoured in blue. Contour plots are stippled when there is at least 75% agreement on the sign of the SLP change (see text for details)

We evaluate the change in SAs using two indices: an area index and a SLP change index. The area of each SA is computed as the area encompassed by the 1020 hPa isobar. Climatologically, subtropical anticyclones are centered towards the eastern flank of the subtropical ocean basins, and shift ~ 5° towards the pole in local summer (Fig. 1, Fig. S1). To account for this seasonality, and target the region of maximum MMM SLP change near the center and along the poleward flank of each SA (Fig. 2), the latitudinal extent of each SLP change index region is 25° S–45° S for DJF and 20° S–40° S for JJA. The longitudinal extent is 112° W–80° W for the SPSA index, 30° W–5° E for the SASA index, and 60° E–105° E for the SISA index (Figs. 5, 6, 8, and 9). A zonal mean SH SLP index is also calculated using the same seasonally varying latitude domain as stated above. The Hadley cell edge is defined here as the latitudinal position of maximum SLP in the SH. The latitude of the maximum zonal mean SLP is calculated by fitting a quadratic to the maximum SLP grid point in the SH and the two grid points either side of it. The latitude at which this quadratic maximizes is then used as the latitude of maximum zonal SLP.

We focus on three main mechanisms identified in previous literature: local diabatic heating, local static stability, and baroclinic instability change. The net diabatic heating for each grid cell’s atmospheric column is calculated as the sum of net longwave radiation, net shortwave radiation, sensible heating, and latent heating following Li et al. (2013). The sensible heating is taken from model’s surface sensible heat flux. The latent heating is converted from precipitation rate: precipitation × *L*, where L = 2.5 × 10^6^ J/kg. The net heating due to longwave and shortwave radiation is taken as the residual going into the atmosphere column (top of the atmosphere—surface).

The static stability of the atmosphere (buoyancy frequency N^2^) is calculated as:

1 $$ N^{2} = \frac{g}{\theta }*\frac{d\theta }{{dz}} $$

where N^2^ > 0 when the stratification is stable, and N^2^ < 0 when the stratification is unstable. In this study N^2^ is vertically averaged between 925 and 300 hPa to make our analysis comparable to that of He et al. (2017).

Following Lu et al. (2008), changes in the conditions promoting baroclinic instability are evaluated with the following metric used in the Phillips Criterion (1954) for baroclinic instability:

2 $$ C \equiv \frac{{f^{2} \left( {u_{500} - u_{lower} } \right)* \Theta }}{{\beta gH\left( {\theta_{500} - \theta_{lower} } \right)}} $$

The conditions promoting baroclinic eddy growth increase with increasing C. The C change can be decomposed into changes due to static stability (C_S_), or the zonal wind component (C_w_).

3 $$ \delta C_{S} = - \frac{{f^{2} \left( {u_{500} - u_{lower} } \right)_{h} *\delta \left( {\theta_{500} - \theta_{lower} } \right)* \Theta }}{{\beta gH\left( {\theta_{500} - \theta_{lower} } \right)^{2}_{h} }} $$

4 $$ \delta C_{w} = \frac{{f^{2} *\delta \left( {u_{500} - u_{lower} } \right)* \Theta }}{{\beta gH\left( {\theta_{500} - \theta_{lower} } \right)_{h} }} $$

where $\delta$ is the difference between RCP8.5/SSP585 and Historical, H is the geometric height of the column—lower level (average between 850 and 1000 hPa) to 500 hPa, and $\Theta$ is a reference potential temperature (300 K). The subscript $h$ refers to the Historical climatology (1950–1999). Given that baroclinic eddy growth is affected more by the lower-level tropospheric baroclinicity (Held and O’Brien 1992), the vertical zonal component of wind shear and potential temperature difference are taken between 500 hPa and the lower level (where the lower level is defined as the averaged between 850 and 1000 hPa).

## Results

### The response of the southern hemisphere subtropical anticyclones in future projections

**Fig. 3:**
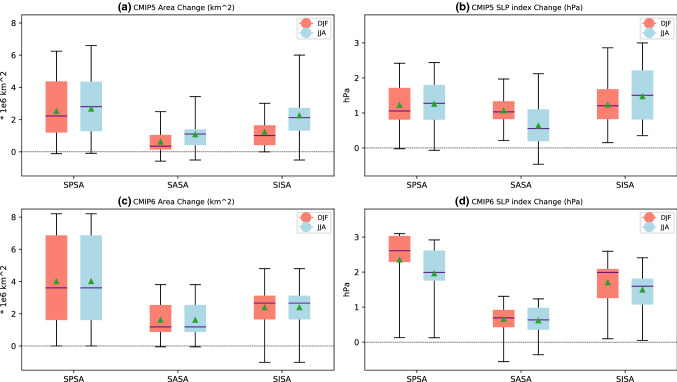
Projected changes in SH SAs **a**, **c** 1020 hPa area (unit: km^2^), and **b**, **d** SLP index (unit: hPa) for **a**, **b** CMIP5 and **c, d** CMIP6. Light red shows DJF and light blue shows JJA. The middle violet lines represent the median change and the green triangles represent the mean change of all the models. The bottom and top of the boxes represent the first and third quartile. The black caps lines represent the minimum and maximum change respectively

We begin by evaluating the area and intensity change of each SH SA between the future projection (2050–2099) and historical (1950–1999) CMIP5 and CMIP6 MMM (Figs. 2, 3). For DJF, SLP decreases along the equatorward flank of the SASA and SPSA, whereas it increases slightly along the equatorward flank of the SISA (Fig. 2a and c). In contrast, all three SH SAs intensify at their center and along their poleward flank in DJF. The 1020 hPa SA area increases for all of the SH SAs and extends poleward. All of the SH SAs also extend further westward during DJF, specifically the SPSA. In the CMIP5 MMM, the SISA area index experiences the greatest relative increase (1.7 × 10^6^ km^2^, a 388% increase) during DJF (Figs. 2, 3, supplementary Table S3). The SPSA also increases significantly in area (2.7 × 10^6^ km^2^, a 161% increase). These two SAs also experience the largest relative increase in DJF area in the CMIP6 MMM (Figs. 2, 3, supplementary Table S4). However, the projected changes in area are not as dramatic in the MMM of the CMIP6 models analyzed. The SPSA increase in area by 3.7 × 10^6^ km^2^ (91% increase), the SASA increases in area by 9 × 10^5^ km^2^ (66% increase), and the SISA increases in area by 1.28 × 10^6^ km^2^ (93% increase) (Figs. 2, 3, supplementary Table S4). It is worth noting that the Historical DJF area is larger to begin with in the CMIP6 MMM compared to the CMIP5 MMM (Fig. 1).

During JJA, SLP at the center and poleward flank of all SH SAs similarly increase, with the latitude of maximum increase shifted between ~ 5–10° N relative to DJF (Fig. 2). Unlike in DJF, the weakening of SLPs on the equatorward flank of the SAs is limited to the far eastern parts of the Pacific and Atlantic basins in JJA. All of the SH SAs increase in area during JJA, extending further towards the pole (Figs. 2 and 3). In CMIP5, the projected JJA changes in area are largest for the SPSA (2 × 10^6^ km^2^, 217% increase), whereas the SISA shows the largest increase in intensity (1.474 hPa) (Figs. 2 and 3, supplementary Table S3). In CMIP6, the SPSA experiences the largest increase in area (3.6 × 10^6^ km^2^, 350% increase), and intensity (1.962 hPa) (Figs. 2 and 3, supplementary Table S4).

During both DJF and JJA, the regions of decreased and increased SLP are associated with cyclonic and anticyclonic circulation anomalies in the 925 hPa wind field, respectively (Fig. 2). In all three SH ocean basins the trade winds (which are a part of the equatorward flank) increase and shift poleward in both seasons (Fig. 2).

**Fig. 4:**
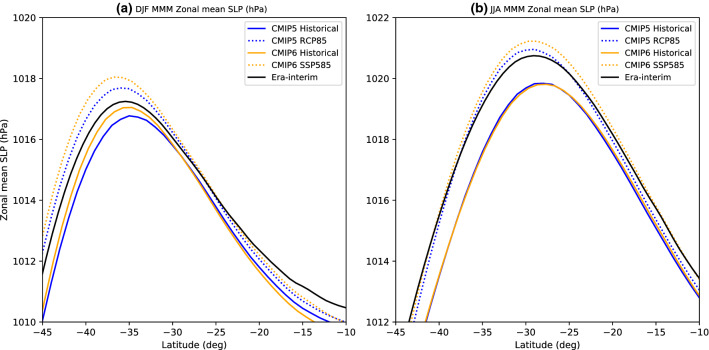
MMM zonal mean SLP (unit: hPa) as a function of the SH latitude for **a** DJF and **b** JJA. The CMIP5 MMM line is plotted in blue, and CMIP6 MMM line is plotted in orange color. The solid line shows the Historical CMIP5 MMM and the dotted line shows the future projections. The black solid line shows Era-Interim climatology of averaged over 1979–2016

In the zonal mean, SLP increases in strength in both seasons and in both the CMIP5 and CMIP6 archive (Fig. 4). The zonal-mean SLP experiences a much larger increase in strength over the subtropics during JJA. The latitude of maximum zonal-mean SLP shifts poleward by 1.2° S in the CMIP5 and 1.3° S in CMIP6 during DJF (Fig. 4). The latitude of zonal mean maximum SLP shifts poleward by 1.2° S in the CMIP5 and 1.2° S in the CMIP6 during JJA (Fig. 4).

**Fig. 5:**
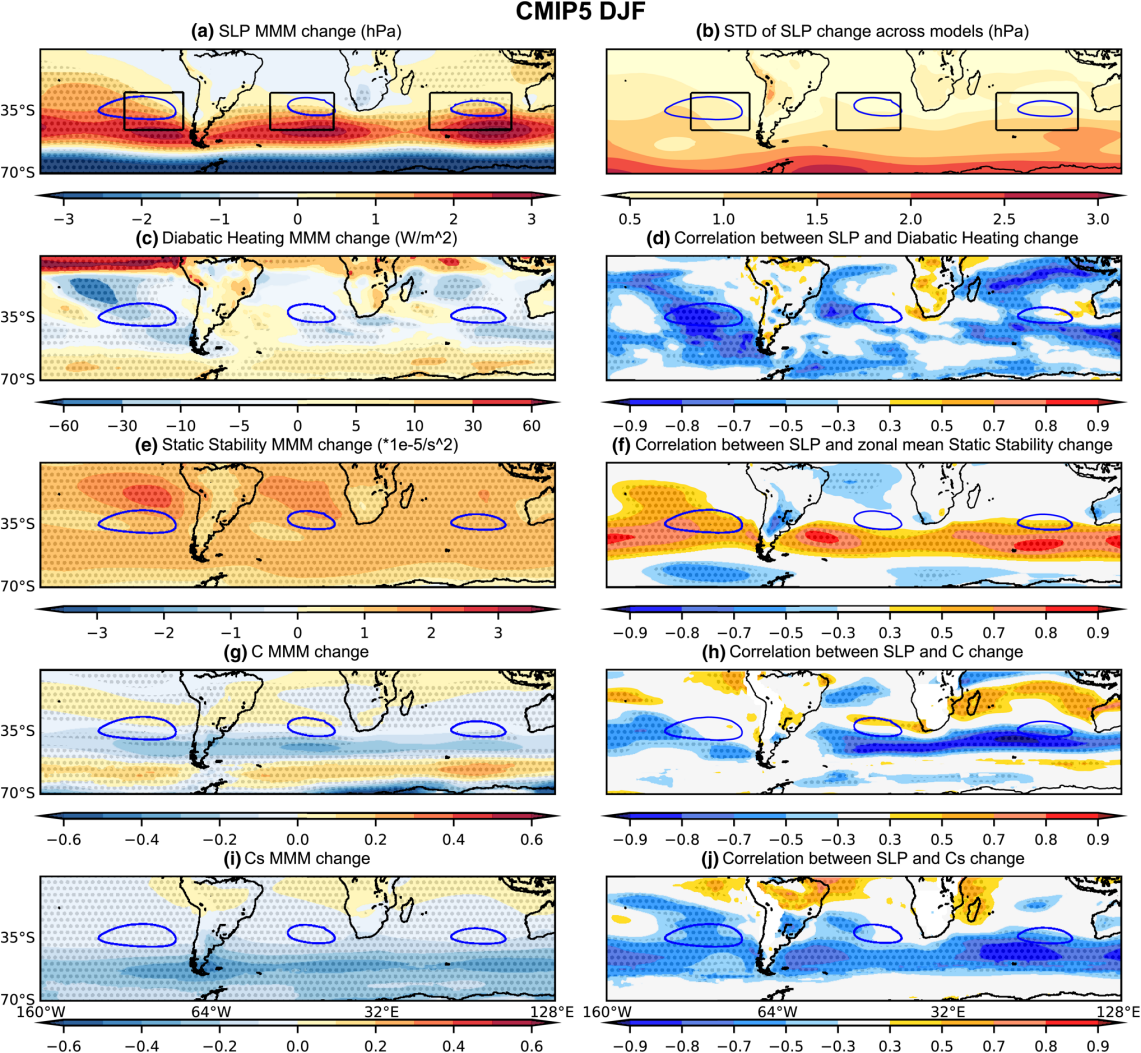
(Left) CMIP5 MMM change (RCP8.5—historical) of the DJF seasonal mean **a** SLP (unit: hPa), **c** Diabatic Heating (unit: W/m^2^), **e** Static Stability (unit: 1 × 10^–5^ s^−2^), **g** C, and **i** C_S_. (Right) **b** Standard deviation of SLP change across models (unit: hPa), and (**d**–**j**) correlation between SLP change and the respective variable change from the corresponding left panel. The 1020 hPa isobar from the MMM projection is contoured in blue. The plots are stippled at 75% agreement for the MMM change plots, and 95% significance level for the correlation plots (see text for details). The index domain for each SA is shown as a contoured black box in **a** and **b**

**Fig. 6:**
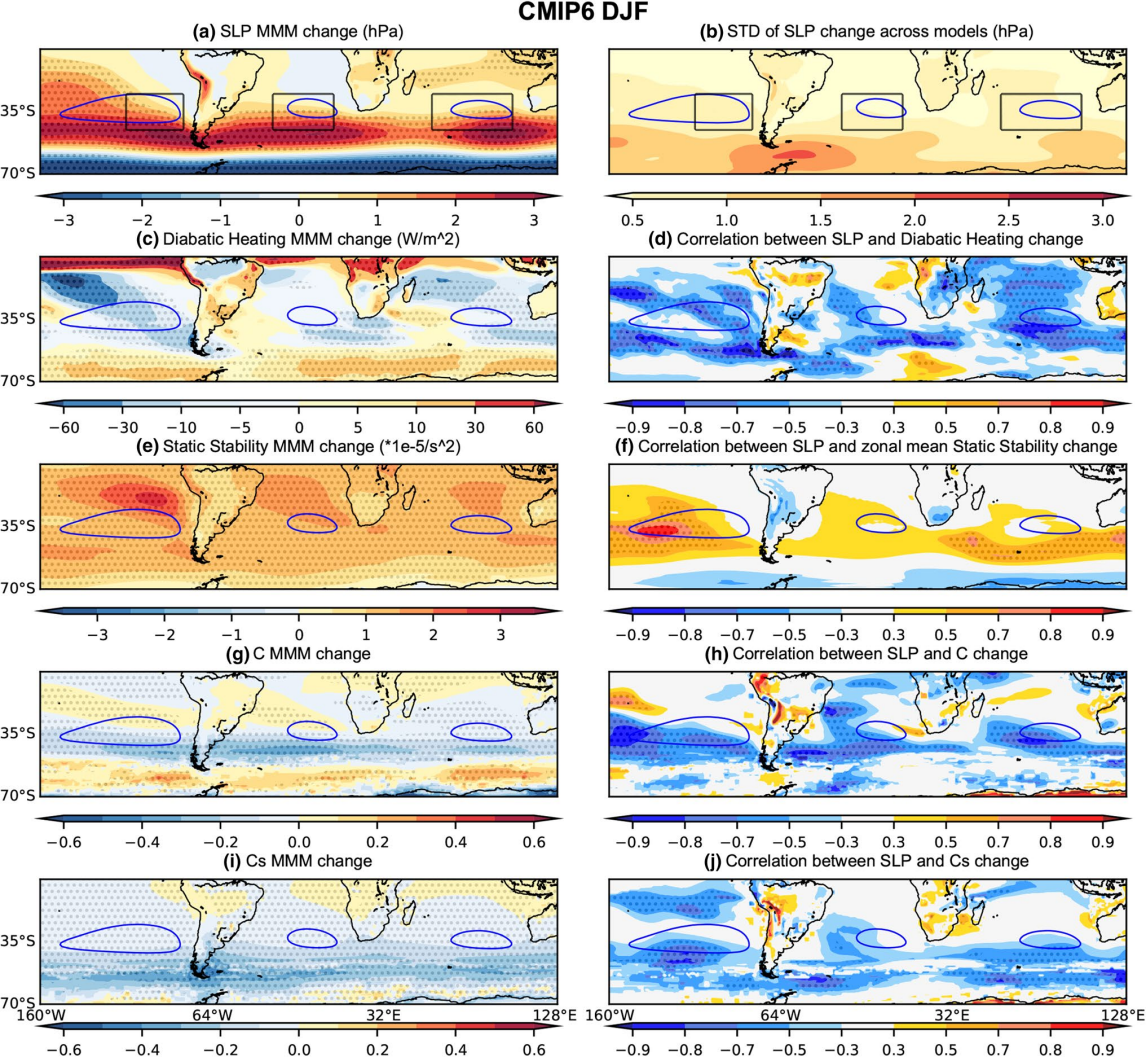
Same as Fig. 5, except for CMIP6 DJF

Although most of the models agree on the sign of the SLP changes associated with the SH SAs in future projections, considerable variability exists across the CMIP5 and CMIP6 models during both DJF and JJA (Figs. 3, 5b, 6b, 8b and 9b). The model spread in the 1020 hPa area change is the largest for the SPSA during both seasons in both CMIP5 and CMIP6 (Fig. 3). The standard deviations in SLP change across both the CMIP5 and CMIP6 models in both seasons shows that the inter-model spread increases from the subtropics to the poleward flank of the SH SAs (Figs. 5b, 6b, 8b and 9b).

### Mechanisms driving multi-model-mean changes and the inter-model spread

#### Diabatic heating changes

**Fig. 7:**
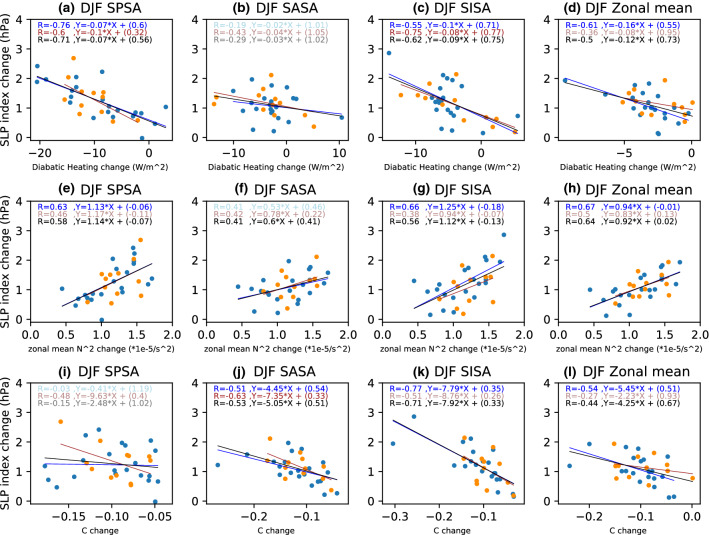
Scatter plots evaluating the relationship between DJF SLP index changes and the corresponding **a**–**d** diabatic heating changes averaged over the respective index regions; **e**–**h** zonal-mean static stability changes averaged over the latitudinal extent of the index regions; **i**–**l** C changes averaged over the respective index regions. **a**, **e**, **i** SPSA; **b**, **f**, **j** SASA; **c**, **g**, **k** SISA and **d**, **g**, **l** the zonal mean SLP index. The CMIP5 archive results are shown in blue and the CMIP6 archive results in orange. The black solid line shows the regression calculated using both the CMIP5 and CMIP6 archives. All correlations exceeding the 95% significance threshold have dark shading

During local summer (DJF), net diabatic heating increases over the SH continents and decreases over the eastern and poleward flanks of each ocean basin in the MMM of both the CMIP5 and CMIP6 future projections (Figs. 5c and 6c). Local decreases in net diabatic heating (enhanced diabatic cooling) are consistent with local increases in SLP and appear to explain a large portion of the model spread—particularly for the SPSA and SISA across the CMIP5 (Fig. 5d) and CMIP6 (Fig. 6d) models (Fig. 7a, c). Variability in the magnitude of SASA changes across both the CMIP5 and CMIP6 model is not as clearly linked to local diabatic heating changes (Fig. 7b). These differences between basins in the extent to which local diabatic heating changes correspond with projected SLP changes across the models (Figs. 5d, 6d and 7) appears to be somewhat linked to the magnitude of the local diabatic heating changes in the MMM and the transition regions (Figs. 5c and 6c). The zonal mean SLP index is only weakly correlated with the projected changes in zonal mean diabatic heating (Fig. 7d).

**Fig. 8:**
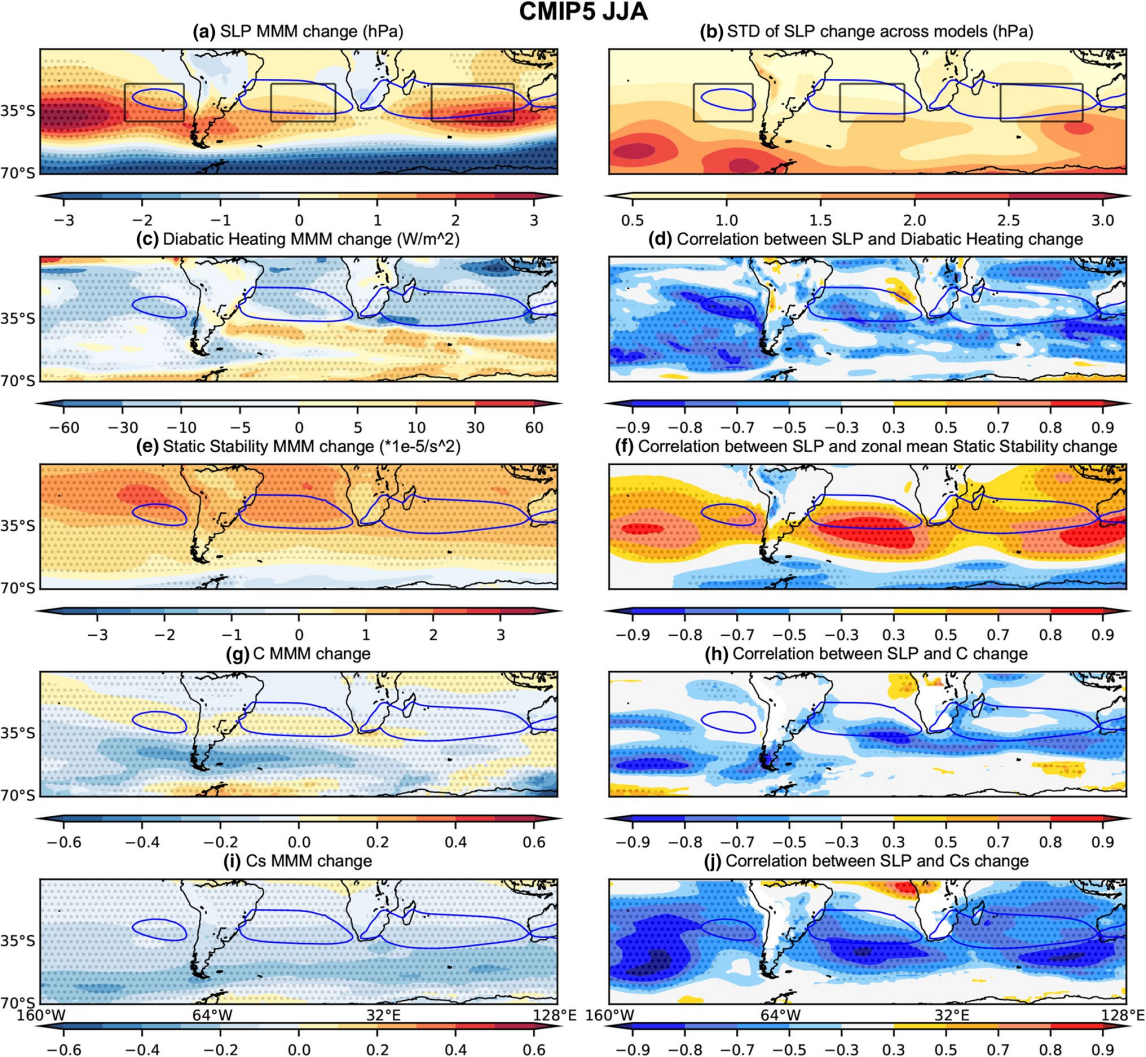
Same as Fig. 5, except for CMIP5 JJA

**Fig. 9:**
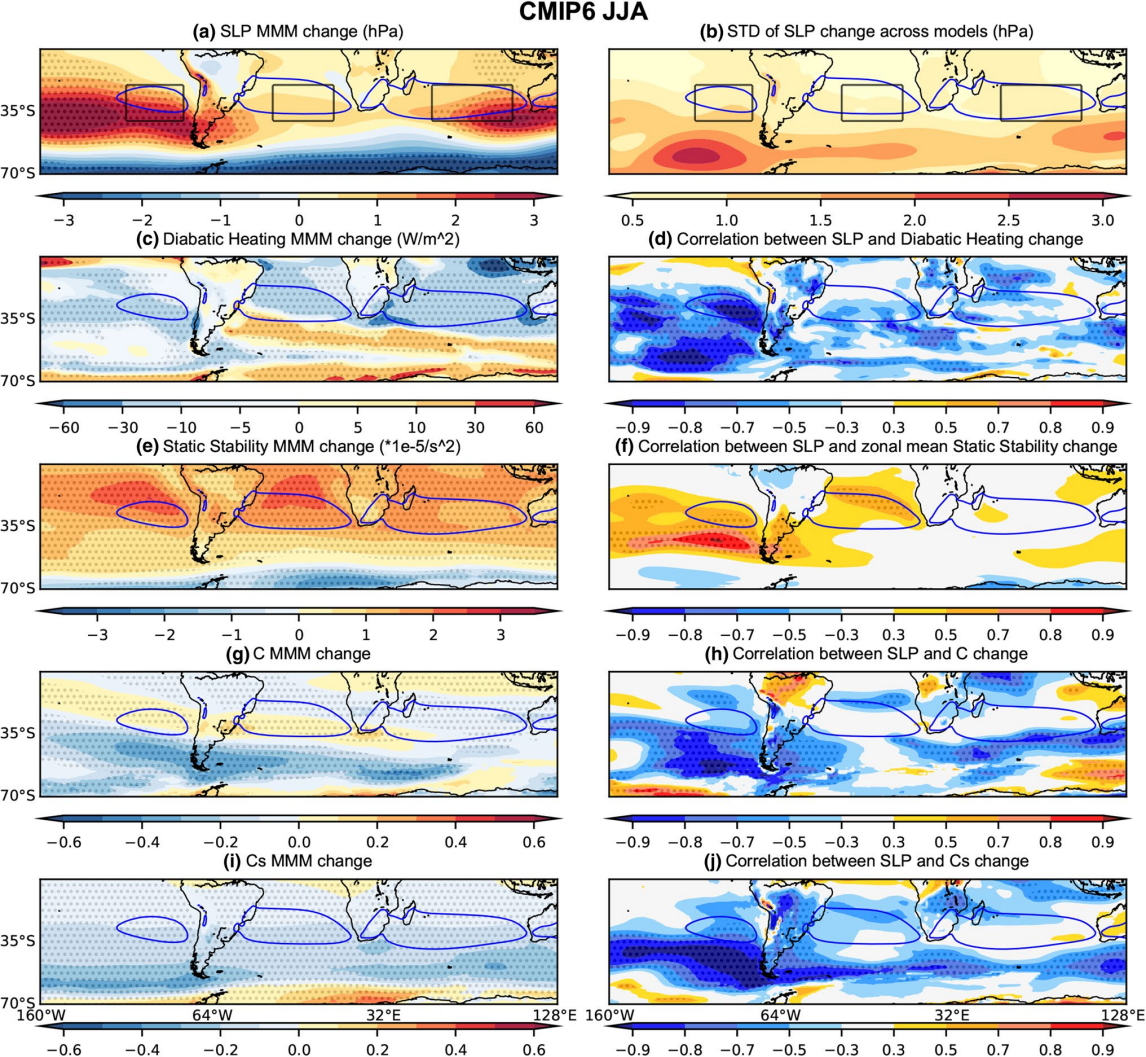
Same as Fig. 5, except for CMIP6 JJA

**Fig. 10:**
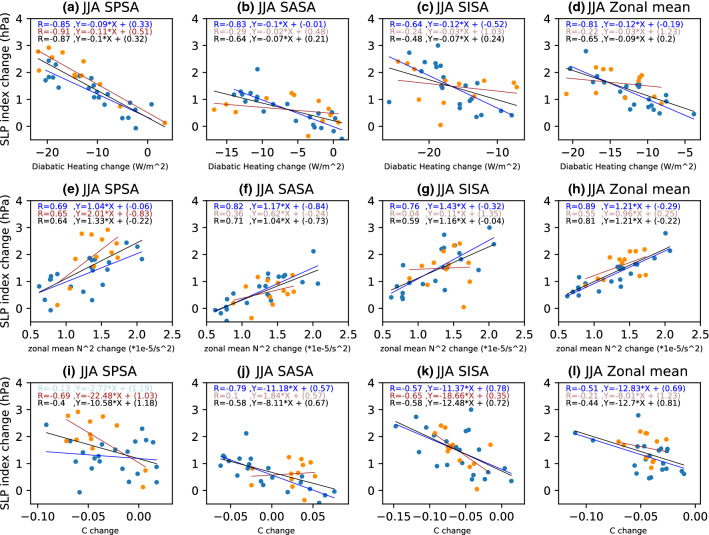
Scatter plots evaluating the relationship between JJA SLP index changes and the corresponding **a**–**d** diabatic heating changes averaged over the respective index regions; **e**–**h** zonal-mean static stability changes averaged over the latitudinal extent of the index regions; **i**–**l** C changes averaged over the respective index regions. **a**, **e**, **i** SPSA; **b**, **f**, **j** SASA; **c**, **g**, **k** SISA and **d**, **g**, **l** the zonal mean SLP index. The CMIP5 archive results are shown in blue and the CMIP6 archive results in orange. The black solid line shows the regression calculated using both the CMIP5 and CMIP6 archives. All correlations exceeding the 95% significance threshold have dark shading

Climatologically, diabatic heating over the continents is weak during austral winter (JJA) and diabatic cooling over the subtropical oceans in the SH reaches its maximum. JJA net diabatic heating decreases over large parts of the SH ocean basins in the MMM CMIP5 and CMIP6 projections, with change maxima occurring somewhat north of the regions of maximum SLP increase (Figs. 8a, c, 9a, c). During JJA, the correlation across models between local diabatic heating changes and changes in the respective SLP indices is strongest for the SPSA (Fig. 10). This correlation between the SPSA index and local diabatic heating changes is stronger in CMIP6 than CMIP5 (Fig. 10), but this could be a function of differences in sample size (an aspect to keep in mind when comparing the CMIP5 versus CMIP6 inter-model spread results). For the SASA and SISA the contrast between CMIP5 and CMIP6 is starker, with less of the model spread in the SASA and SISA change explained by diabatic heating changes. Looking at the spatial patterns in the local correlation between SLP change and diabatic heating change across the models, the spread in diabatic heating changes across the CMIP5 models appears to be more strongly associated with local SLP changes in the SASA and SISA regions than in CMIP6, while strong correlations are seen in the SPSA regions in both CMIP5 and CMIP6 (Figs. 8d and 9d). The zonal mean SLP change index is also significantly correlated (− 0.81) during JJA with zonal mean diabatic heating change in the CMIP5 archive, whereas the correlation weakens across the CMIP6 models (− 0.22) (Fig. 10).

#### Static stability changes

**Fig. 11:**
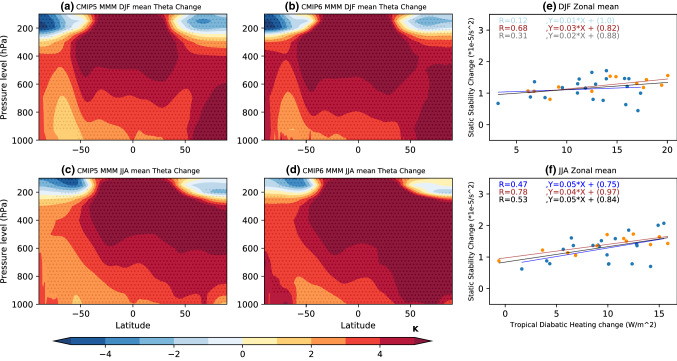
Future projection—historical **a**, **c** CMIP5 and **b**, **d** CMIP6 MMM potential temperature change (K) shown for **a**, **b** DJF, and **c**, **d** JJA. Contour plots are stippled at 75% significance level (see text for details). **e**, **f** Scatter plots evaluating the relationship between zonal mean tropical (10° S–10° N) diabatic heating change and zonal mean (vertically averaged between 925 and 300 hPa) subtropical (25° S–45° S for DJF and 20° S–40° S for JJA) static stability (N^2^) change for **e** DJF and **f** JJA. The CMIP5 archive results are shown in blue and the CMIP6 archive results in orange. The black solid line shows the regression calculated using both the CMIP5 and CMIP6 archives. All correlations exceeding the 95% significance threshold have dark shading in scatter plots

**Fig. 12:**
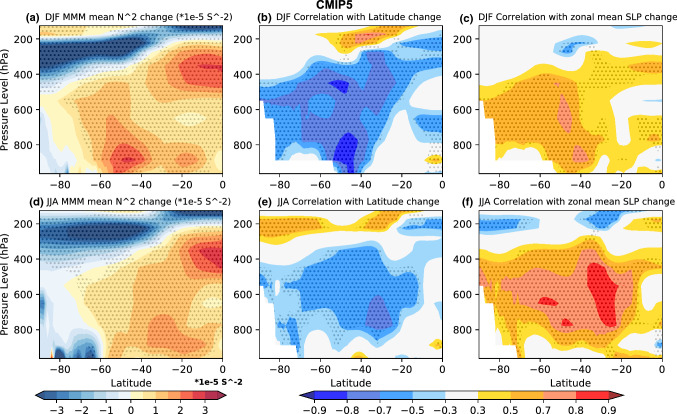
The CMIP5 zonal mean static stability changes (unit: 1 × 10^–5^ s^−2^) between future projection and Historical MMM shown in for **a** DJF, and **d** JJA. The correlation across models between the change in SH Hadley cell edge latitude and static stability is shown in **b** for DJF, and **e** JJA. The correlation between the zonal mean SLP index change and static stability change is shown in **c** for DJF, and **f** JJA. The plots are stippled at 75% significance level for change, and 95% significance level for correlation (see text for details)

**Fig. 13:**
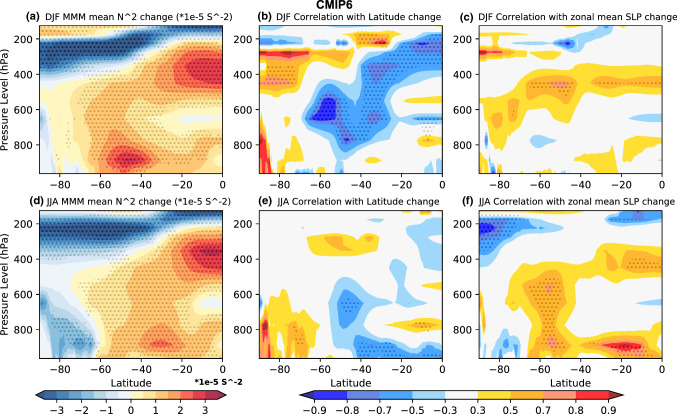
Same as Fig. 12, except for CMIP6

With the exception of the high latitude NH during DJF, enhanced warming of the upper to mid troposphere leads to MMM static stability increases (Figs. 11, 12, 13). Static stability averaged between 925 and 300 hPa increases over all SH ocean basins in the MMM of future projections during both seasons (Figs. 5e, 6e, 8e, 9e), with the largest static stability increases occurring along the equatorward flank of the SPSA and SASA.

In the MMM these static stability changes are largely the result of enhanced tropical latent heat release raising temperatures in the upper tropical troposphere (Fig. 11) (Lu et al. 2008; Ma et al. 2012). When it comes to the inter-model spread in projected zonal-mean subtropical static stability changes, the connection with projected changes in vertically integrated zonal mean tropical diabatic heating (averaged between 10° N and 10° S) is weak for DJF (with the exception of the CMIP6 sample) (Fig. 11e), but it is significant for JJA (Fig. 11f). This result points to the variability across models in subtropical lower tropospheric warming playing an equally important role in dictating the nature of subtropical static stability changes as variability in tropical diabatic heating, particularly for DJF.

He et al. (2017) attribute the DJF SLP weakening seen along the equatorward flank of the SAs (Figs. 5a, 6a) to the zonal-mean increase in static stability via the MASC mechanism (Ma et al. 2012). Interestingly however, differences in zonal-mean static stability changes across the models have no significant negative relationship with the spread in DJF SLP changes along the equatorward flank (Figs. 5f, 6f). Along the poleward flank on the other hand, the DJF spread in zonal-mean static stability changes are positively (rather than negatively) correlated with the spread in CMIP5 (Fig. 5f) and CMIP6 (Fig. 6f) SLP change. Similarly, for JJA a positive correlation is seen between subtropical SLP change and zonal-mean tropospheric static stability change across the models (Figs. 8f, 9f). Note that the correlations shown in Figs. 5e, 6e, 8e and 9e are between the zonal-mean static stability and local SLP changes at every grid point. This is done because static stability changes can force the circulation change or be forced by the circulation change (He et al. 2017). Given that the high correlations seen between subtropical zonal-mean static stability changes and SLP changes are opposite in sign to that expected based on the MASC mechanism described He et al. (2017), there has to be a different process explaining this relationship (Figs. 7 and 10). It should be noted that while the model spread in static stability changes and hence the MASC mechanism do not appear to explain the model spread in SLP responses along the equatorward flank of the SAs, our analysis does not rule it out as the mechanism driving the MMM weakening (Figs. 5a, e, 6a, e, 8a, e, 9a, e) as found by He et al. (2017).

#### Conditions promoting baroclinic instability

The conditions promoting baroclinic instability are evaluated using changes in the Phillips Criterion metric for eddy growth, C, which occur due to changes in both zonal wind shear and static stability (500 hPa—lower level). During DJF, C moderately increases in parts of the equatorward flank of the SPSA and SASA (Figs. 5g and 6g). However, C decreases along the poleward flank of all SH SAs (Figs. 5g and 6g). These decreases in C are consistent with the amplified SLP changes along the poleward flank of the SAs, as decreases in the conditions promoting eddy growth correspond with regions of SLP increase. Moreover, across the CMIP5 (Fig. 5h) and CMIP6 (Fig. 6h) models, this mechanism is strongly related to the spread in DJF SLP responses along the poleward flank of the SAs. When looking at the SA intensity indices (Fig. 7), which capture changes across the whole SA region rather than just the poleward flank, the correlation between these variables across models is lower than along the poleward flank (Figs. 5h and 6h). Nevertheless, the relationship with C changes is stronger over the SASA and SISA (Fig. 7). During JJA, there are similarly decreases in C along the poleward flank of all SH SAs (Figs. 8g and 9g). When looking at the JJA SA intensity indices (Fig. 10), the correlation between projected SLP and C change is also significant, specially over the SASA and SISA, but once again the index captures changes across the whole SA region rather than just the poleward flank such that the correlation between these variables across models is lower than what is seen along the poleward flank in the spatial correlation plots (Figs. 8h and 9h).

Interestingly, the Phillips Criterion metric for eddy growth due to only static stability change (Cs) explains a significant portion of inter-model variability in SLP change both near the center and along the poleward flank of the SAs during both seasons, particularly in the CMIP5 archive (Figs. 5j and 8j). Due to changes in the static stability of the troposphere, baroclinic eddy growth decreases in the SH subtropical and midlatitude region during both seasons (Figs. 5i, 6i, 8i, and 9i). During DJF, the Cs decrease is maximum in a mid-latitude band centered between 40°–50° S, which is largely zonally symmetric (Figs. 5i and 6i). The negative correlation between SLP change and Cs change along the poleward flank of SH SAs increases in JJA in the CMIP5 archive (Fig. 8j). However, the correlation is weaker (relatively speaking) over the SASA and SISA in the CMIP6 archive (Fig. 9j). The Phillips Criterion metric for eddy growth due to only zonal wind change (Cw) partially explains the SLP changes over the poleward flanks of the SASA and SPSA during both seasons, particularly in the CMIP6 archive (supplementary Fig. S2 and S3), but positive MMM Cw increases in the center and equatorward flank of the SAs acts to counter the static stability induced reductions in C and hence SLP increases. This could be one reason why the MMM SLPs increase more along the poleward flanks of the SAs than at their centers (Figs. 5a, 6a, 8a and 9a).

The CMIP inter-model spread in the maximum SLP latitude and zonal mean SLP index change appears to be largely linked to changes in the conditions prompting baroclinic eddy growth in the SH. The changes in the conditions promoting baroclinic eddy growth are in turn mostly driven by tropospheric static stability changes. SH zonal mean static stability increases most in the upper tropical troposphere and lower midlatitude troposphere (Figs. 12a, d, 13a, d). The correlation across the models shows that the latitude of maximum SLP shifts poleward with increased tropospheric static stability in future projections (Figs. 12b, e, 13b, e). The correlation across the models also shows that the zonal mean SLP increases over the subtropics with increased tropospheric static stability during both seasons (Figs. 12c, f, 13c, f).

## Discussion and conclusions

This study assesses both the local summer (DJF) and winter (JJA) projected changes in the SH SAs in two future scenarios from the CMIP5 and CMIP6 archives. The mechanisms giving rise to these SLP changes in the MMM, as well as the spread in the response across models have been investigated.

SLP associated with SH SAs increases during both seasons in the MMM of the CMIP5 and CMIP6 future projections, with the exception of the equatorward flanks of the SASA and SPSA (particularly during DJF). The area and intensity of all SH SAs increases in future projection and extends poleward. Although most of the CMIP5 and CMIP6 models agree on the sign of SLP changes, there is a large inter-model spread in the magnitude of this change, particularly along the poleward flank where the largest MMM changes occur. In this study we have investigated the role of local diabatic heating, zonal-mean tropical diabatic heating, zonal-mean subtropical static stability, and local changes in the conditions promoting baroclinic instability as potential mechanisms that can explain this inter-model spread in subtropical SLP change.

Local, MMM diabatic heating decreases substantially over the subtropical ocean basins in both seasons, with the strongest changes in the Atlantic and Indian Oceans occurring during JJA. These decreases in local diabatic heating are consistent with local SLP increases over the SH subtropical Ocean. The inter-model spread in local diabatic heating accounts for a considerable amount of the inter-model spread in SPSA strength changes during both DJF and JJA (for both the center of the high as defined by the SPSA intensity index and the broader region evident in the spatial correlation maps). In the Atlantic and Indian Oceans on the other hand, regions of strong correlation between the inter-model spread in local diabatic heating and local SLP changes are more regionally confined and differ more between the CMIP5 and CMIP6 samples and seasons.

SH subtropical tropospheric static stability changes substantially in future projections in both the CMIP5 and CMIP6 archive. In the MMM, increased latent heating in the upper tropical troposphere appears to be the primary driver of the static stability increases over the subtropics in both seasons. The inter-model spread in projected tropical diabatic heating changes however only explains a portion of the inter-model spread in subtropical static stability, suggesting that the model spread in subtropical-to-midlatitude lower tropospheric heating plays a comparable role in the spread in static stability changes.

The MMM SLP weakening seen on the equatorward flank of the SH SAs is consistent with the MMM zonal-mean static stability increase via the MASC mechanism. There is however no significant relationship between the model differences in zonal-mean static stability changes and the extent to which SLP weakens in their equatorward flank. On the contrary, the correlation across models between zonal-mean static stability change and local SLP change shows significant positive correlations over the center and poleward flank during both DJF and JJA. This positive, rather than negative, correlation suggest that a mechanism related to zonal-mean static stability changes other than the MASC mechanism is impacting the SAs along their poleward flank and therefore, based on the results of the previous studies cited in Sect. 1, the influence of static stability changes on the conditions promoting baroclinic instability is investigated.

The Phillips Criterion metric is used to evaluate the contribution of changes in both static stability and zonal wind shear towards changes in the conditions promoting baroclinic eddy growth. Projected changes in the Phillips Criterion metric, C, indicate a decrease in the conditions promoting baroclinic instability along poleward flank of the SH SAs during both seasons in both the CMIP5 and CMIP6 MMM, while slight increases are seen in the center/equatorward flank to varying degrees depending on CMIP sample and season. The MMM decreases in C along the poleward flank of the highs help explain the amplified MMM SLP changes in these regions. Similarly, the inter-model spread in projected local changes in C accounts for a large amount of the spread in SLP changes in several regions along the poleward flanks of the SH SAs.

When focusing on the relative contribution of static stability changes versus zonal wind shear changes to the Phillips Criterion metric, the contribution of MMM static stability changes to Cs changes alone supports a strengthening of the SAs on both their poleward and equatorward flanks—although more so on the poleward flank. Turning to the inter-model spread, high correlations are seen across the models between Cs and SLP along both the poleward flanks, and to varying degrees the center of the SAs. It is the contribution of positive zonal wind shear changes along the equatorward flanks of the SAs that acts to increase C in these regions, potentially acting in encourage eddy growth and mute SLP increases in these regions. Along the poleward flanks on the other hand, both Cs and Cw changes act to suppress eddy grow and increase SLP. The SH Hadley cell edge (latitude of maximum SLP) shifts poleward in both seasons in the CMIP5 and CMIP6 future projections analyzed.

In summary, our results suggest that the model spread in SH SLP projections results from a combination of the spread in local diabatic heating and zonal-mean static stability changes. The zonal-mean static stability changes drive the spread in the SAs through their influence on the conditions promoting baroclinic instability rather than the MASC mechanism. Overall, the results presented in this study suggest that differences in warming between the tropical upper troposphere and the subtropical to mid-latitude lower troposphere in the SH, via their influence on SH tropospheric static stability, will largely determine the fate of the SH SAs over the coming century.

## Electronic supplementary material

Below is the link to the electronic supplementary material.

Supplementary file1 (DOCX 981 kb)
