# Supplementary material for: How will southern hemisphere subtropical anticyclones respond to global warming? Mechanisms and seasonality in CMIP5 and CMIP6 model projections
Source: Clim Dyn. 2020 May 10;55(3):703–18. doi: 10.1007/s00382-020-05290-7 (PMC7370984; doi:10.1007/s00382-020-05290-7)
Supplement: Supplementary file 1 — Supplementary file1 (DOCX 981 kb) [file 382_2020_5290_MOESM1_ESM.docx]

**Supplementary**

**Tables:**

***Table S1:*** *List of CMIP5 models and realizations*

| **SL NO.** | **Model Name** | **Total Realizations** | **SL NO.** | **Model Name** | **Total Realizations** |
| --- | --- | --- | --- | --- | --- |
| 1 | ACCESS1-0 | 1 | 12 | GISS-E2-H-CC | 1 |
| 2 | ACCESS1-3 | 3 | 13 | GISS-E2-H | 6 |
| 3 | CanESM2 | 5 | 14 | GISS-E2-R-CC | 1 |
| 4 | CCSM4 | 5 | 15 | GISS-E2-R | 6 |
| 5 | CMCC-CM | 1 | 16 | inmcm4 | 1 |
| 6 | CMCC-CMS | 1 | 17 | IPSL-CM5A-LR | 6 |
| 7 | CNRM-CM5 | 10 | 18 | IPSL-CM5A-MR | 3 |
| 8 | CSIRO-Mk3-6-0 | 10 | 19 | IPSL-CM5B-LR | 1 |
| 9 | GFDL-CM3 | 5 | 20 | MPI-ESM-LR | 3 |
| 10 | GFDL-ESM2G | 1 | 21 | MPI-ESM-MR | 3 |
| 11 | GFDL-ESM2M | 1 |  |  |  |

***Table S2****: List of CMIP6 models and realizations*

| **SL NO.** | **Model Name** | **Total Realizations** | **SL NO.** | **Model Name** | **Total Realizations** |
| --- | --- | --- | --- | --- | --- |
| 1 | BCC-CSM2-MR | 3 | 7 | MIROC-ES2L | 3 |
| 2 | CAMS-CSM1-0 | 2 | 8 | MIROC6 | 10 |
| 3 | CanESM5 | 50 | 9 | UKESM1-0-LL | 9 |
| 4 | CNRM-CM6-1 | 19 | 10 | MRI-ESM2-0 | 5 |
| 5 | CNRM-ESM2-1 | 5 | 11 | CESM2-WACCM | 3 |
| 6 | IPSL-CM6A-LR | 32 | 12 | CESM2 | 11 |

***Table S3:*** *CMIP5 SH SAs 1020 hPa area and intensity change (RCP8.5-Historical) in DJF and JJA*

| **Properties** | **SA** | **DJF** | | **JJA** | |
| --- | --- | --- | --- | --- | --- |
|  |  | Historical | Change  (RCP8.5-Historical) | Historical | Change  (RCP8.5-Historical) |
|  | SPSA | *1695744.38* | *2724811.97 (160.68%)* | *911112.33* | *1978486.83 (217.2%)* |
| **Area** | SASA | *1381039.45* | *832733.60 (60.30%)* | *9394343.94* | *891181.50 (9.48%)* |
| (Km^2^) | SISA | *427408.73* | *1658194.60 (387.96%)* | *13018493.93* | *2219400.19 (17.04%)* |
|  | SPSA | *1018.11* | *1.226* | *1017.92* | *1.257* |
| **Intensity** | SASA | *1017.10* | *1.065* | *1020.72* | *0.636* |
| (hPa) | SISA | *1016.73* | *1.229* | *1020.34* | *1.474* |

***Table S4:*** *CMIP6 SH SAs 1020 hPa area and intensity change (SSP585-Historical) in DJF and JJA*

| **Properties** | **SA** | **DJF** | | **JJA** | |
| --- | --- | --- | --- | --- | --- |
|  |  | Historical | Change  (SSP585-Historical) | Historical | Change  (SSP585-Historical) |
|  | SPSA | *4054720.82* | *3676823.52 (90.68%)* | *1022797.59* | *3579651.58 (349.99%)* |
| **Area** | SASA | *1380874.72* | *915104.30 (66.27%)* | *9571690.92* | *1199741.37 (12.53%)* |
| (Km^2^) | SISA | *1377372.37* | *1278002.95 (92.79%)* | *12755285.75* | *2834827.65 (22.22%)* |
|  | SPSA | *1019.16* | *1.321* | *1017.93* | *1.962* |
| **Intensity** | SASA | *1017.39* | *1.174* | *1021.10* | *0.617* |
| (hPa) | SISA | *1017.38* | *1.082* | *1020.81* | *1.494* |

**Figures**

**
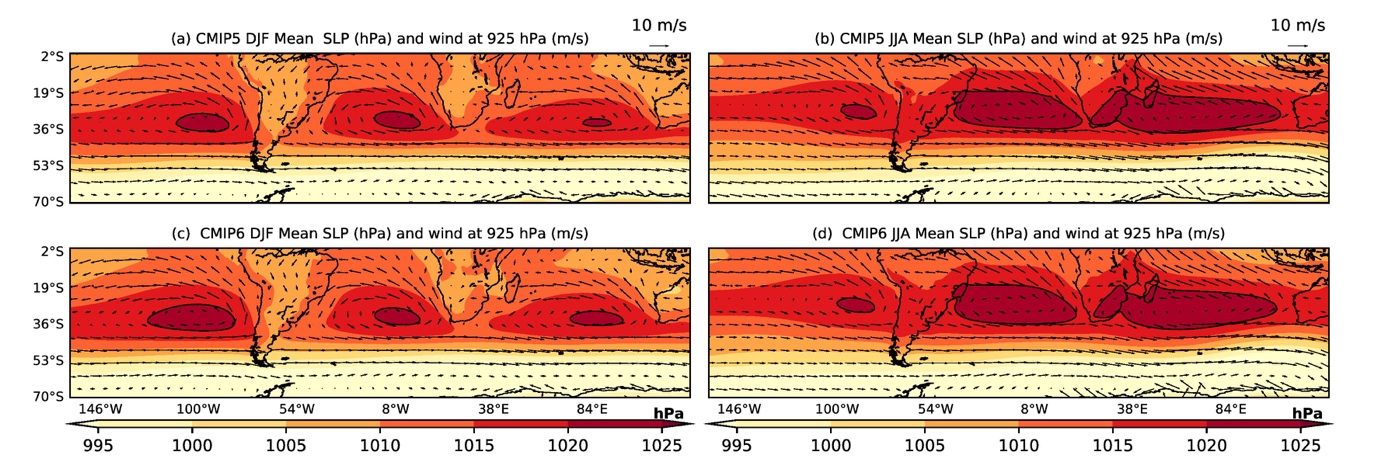
**

**Fig. S1:** CMIP5 and CMIP6 climatology of (a/c) DJF and (b/d) JJA seasonal mean SLP (shaded) (unit: hPa), wind at 925hPa (vector) (unit: m/s).

**
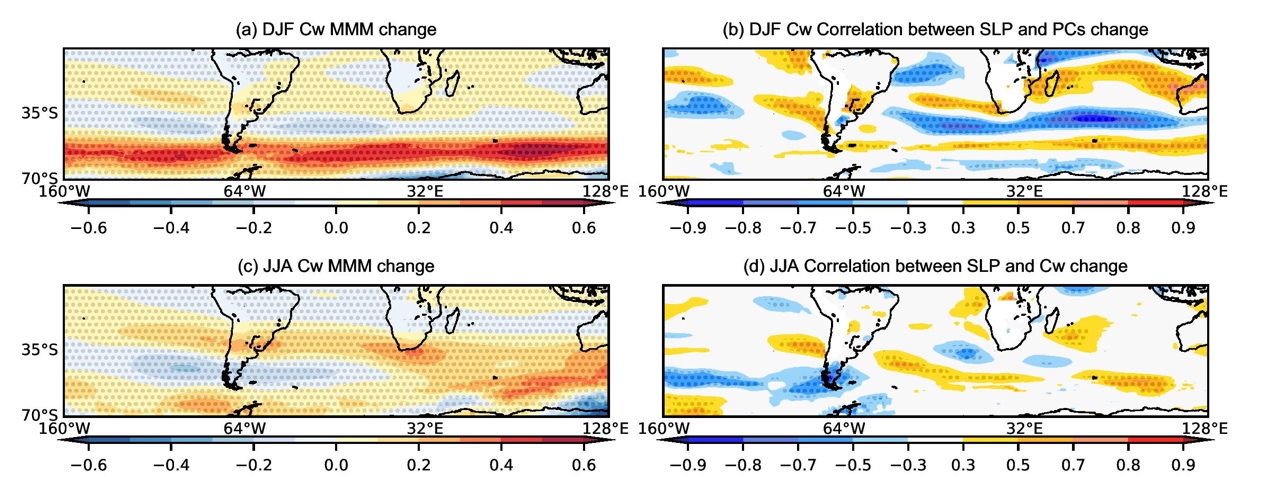
**

**Fig. S2** The CMIP5 Cw change between RCP8.5 and Historical MMM shown in (a) for DJF, and (c) for JJA. The local correlation between SLP change and Cw change is shown in (b) for DJF, and (d) JJA. The 1020hPa area of CMIP5 Historical MMM is contoured in black.

**
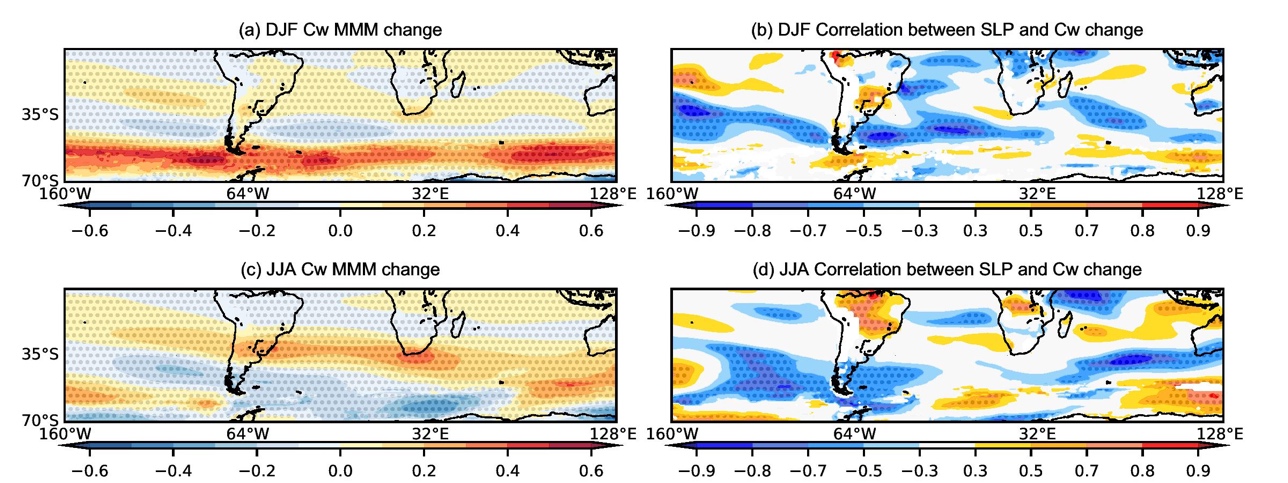
**

**Fig. S3** The CMIP6 Cw change between SSP585 and Historical MMM shown in (a) for DJF, and (c) for JJA. The local correlation between SLP change and Cw change is shown in (b) for DJF, and (d) JJA. The 1020 hPa area of CMIP5 Historical MMM is contoured in black.
